# Validation of the 21st Century Skills Assessment Scale for public health students in Thailand: a methodological study

**DOI:** 10.3352/jeehp.2024.21.37

**Published:** 2024-12-10

**Authors:** Suphawadee Panthumas, Kaung Zaw, Wirin Kittipichai

**Affiliations:** Department of Family Health, Faculty of Public Health, Mahidol University, Bangkok, Thailand; Hallym University, Korea

**Keywords:** Statistical factor analysis, Public health, Student, Educational measurement, Self-assessment

## Abstract

**Purpose:**

This study aimed to develop and validate the 21st Century Skills Assessment Scale (21CSAS) for Thai public health (PH) undergraduate students using the Partnership for 21st Century Skills framework.

**Methods:**

A cross-sectional survey was conducted among 727 first- to fourth-year PH undergraduate students from 4 autonomous universities in Thailand. Data were collected using self-administered questionnaires between January and March 2023. Exploratory factor analysis (EFA) was used to explore the underlying dimensions of 21CSAS, while confirmatory factor analysis (CFA) was conducted to test the hypothesized factor structure using Mplus software (Muthén & Muthén). Reliability and item discrimination were assessed using Cronbach’s α and the corrected item-total correlation, respectively.

**Results:**

EFA performed on a dataset of 300 students revealed a 20-item scale with a 6-factor structure: (1) creativity and innovation; (2) critical thinking and problem-solving; (3) information, media, and technology; (4) communication and collaboration; (5) initiative and self-direction; and (6) social and cross-cultural skills. The rotated eigenvalues ranged from 2.12 to 1.73. CFA performed on another dataset of 427 students confirmed a good model fit (*χ*^2^/degrees of freedom=2.67, comparative fit index=0.93, Tucker-Lewis index=0.91, root mean square error of approximation=0.06, standardized root mean square residual=0.06), explaining 34%–71% of variance in the items. Item loadings ranged from 0.58 to 0.84. The 21CSAS had a Cronbach’s α of 0.92.

**Conclusion:**

The 21CSAS proved be a valid and reliable tool for assessing 21st century skills among Thai PH undergraduate students. These findings provide insights for educational system to inform policy, practice, and research regarding 21st-century skills among undergraduate students.

## Graphical abstract

[Fig f2-jeehp-21-37]

## Introduction

### Background/rationale

In today’s rapidly evolving world, the Partnership for 21st Century Learning (P21) framework emphasizes essential skills for success in academic and professional settings [1]. Integrating these competencies with traditional subjects prepares students to apply knowledge in real-world scenarios, increasing their readiness for future challenges [2]. Supported by UNESCO (United Nations Educational, Scientific, and Cultural Organization), this educational shift calls for innovative policies, curriculum reforms, and a broader understanding of education’s role in fostering global citizenship, technological literacy, and ethical leadership [3].

Thailand’s Community Health Professional Council has outlined competencies aligned with 21st-century skills, reinforcing abilities such as critical thinking, communication, and collaboration essential for tackling public health challenges. These competencies highlight the need for research focusing on bachelor of public health programs in Thailand, supporting skills required for modern public health roles.

Measuring 21st-century skills provides insights into student capabilities, influencing course design and encouraging the teaching of these skills [4]. However, assessing these skills is challenging, particularly in tracking student progress [5]. The emphasis on embedding these skills into curricula has increased the need for reliable and valid assessment instruments. Measuring creativity, critical thinking, and collaboration requires clear frameworks that translate these abstract concepts into measurable competencies. Educational research has increasingly employed methods such as factor analysis to ensure validity and reliability [6]. Recent studies have developed diverse scales to measure 21st-century skills, emphasizing the need for context-specific tools tailored to various disciplines and student populations [7-9].

Despite this progress, there remains a gap in assessing 21st-century skills among public health undergraduates. This study addresses this gap by applying the P21 framework to a culturally specific public health context, emphasizing the unique contributions of the 21st Century Skills Assessment Scale (21CSAS). Unlike traditional health education assessments that focus on knowledge retention, 21CSAS emphasizes critical competencies such as communication and creativity, which are crucial for culturally adaptive health practices. Tailored assessments that incorporate community-specific beliefs make 21CSAS particularly valuable in diverse public health settings.

### Objectives

The purpose of this study was to develop and validate a 21st-century skills assessment scale for Thai public health undergraduates using the P21 framework.

## Methods

### Ethics statement

The study was approved by the Human Research Ethics Committee of the Institute for Population and Social Research, Mahidol University (COA no., 2022/09-187) on December 14, 2022. Informed consent was acquired from all participants.

### Study design

This study was a cross-sectional survey to validate an assessment tool.

### Setting

This study was conducted in the setting of bachelor of public health programs of Thai autonomous universities accredited by the Community Public Health Council [10]. Data were collected between January and March 2023.

### Participants

A 2-stage sampling method was used. In the first stage, 12 autonomous universities with accredited bachelor of public health programs were grouped into 4 regions. In the second stage, one university from each region was selected through simple random sampling. Data were collected from first to fourth-year students in good health during the 2022 academic year. Between 180–200 eligible students were recruited from each university.

### Data sources/measurement

The 21CSAS was developed based on the P21 framework, focusing on 3 key domains: (1) learning and innovation skills (LI), which are critical for students to thrive in increasingly complex life and work environments. This domain includes creativity, critical thinking, communication, and collaboration; (2) information, media, and technology skills (IMT), which refer to students’ abilities to access, analyze, evaluate, and produce information and media effectively, accurately, and ethically; and (3) life and career skills (LC), which encompass work-life balance, professionalism, ethics, and the ability to work effectively across cultural differences.

The preliminary 21CSAS consisted of 21 items drafted from the definitions of the 3 domains with content validity confirmed by 3 experts, resulting in a scale content validity index (CVI) of 0.91 and item CVIs from 0.8 to 1. Face validity was validated through feedback from 30 public health undergraduates.

The questionnaire, consisting of 21 items (LI: 10, IMT: 5, LC: 6), was rated on a 5-point scale (1=strongly disagree to 5=strongly agree) and administered once to 780 participants. Of these, 727 responses (93.21%) were completed and deemed suitable for analysis. The dataset was divided into 2 subsets: one with 300 participants for exploratory factor analysis (EFA) and another with 427 participants for confirmatory factor analysis (CFA) (Dataset 1).

### Variables

Demographic variables included gender and academic year. The 21CSAS consisted of 21 items, with latent variables to be identified as part of the analysis.

### Bias

Self-reports can be biased, such as when participants provide socially acceptable answers (social desirability bias) or struggle to recall past behaviors (recall bias). Differences in understanding or motivation may also cause inconsistencies. To reduce biases, the following measures were taken: ensuring anonymity, using validated scales, and asking neutral questions. Nonetheless, the results should be interpreted carefully.

### Study size

The sample size calculation followed the N:q rule of thumb, where N represents the number of cases, and q denotes the number of model parameters [6]. For this study, a recommended ratio of 15–20:1 for 20 parameters, a minimum of 600 participants was required across 2 datasets (300 each) for the factor analysis (EFA and CFA) utilized in this study. To account for potential data loss, 30% more participants were included, resulting in a final sample size of 780.

### Statistical methods

Mplus software ver. 8.10 (Muthén & Muthén; license no., SABC80022922) with the maximum likelihood robust estimator was used to perform EFA using oblique rotation with the Geomin method. Additionally, CFA was conducted. The quality of fit of the models was assessed by meeting reference values of good quality indicators (Table 1) [11].

## Results

### Participants

The sample comprised 727 undergraduate students enrolled in bachelor of public health programs. The majority (87.8%, n=638) were women. The distribution by academic year was as follows: 27.1% (n=197) third-year, 26.1% (n=190) first-year, 24.8% (n=180) second-year, and 22% (n=160) fourth-year students.

### Main results

#### Exploratory factor analysis

EFA was performed on an initial 21CSAS using a dataset of 300 participants. Initially containing 21 items, 1 item was removed, leaving a final version of 20 items. The EFA revealed a 6-factor structure with good model fit (Tables 1–3). The 6 identified dimensions are as follows: (1) Creativity and innovation skills (CI): Refers to the ability to proactively identify problems, apply knowledge to real-world situations, and develop innovative solutions through the design thinking process, which includes empathy, defining the problem, ideation, prototyping, and testing (3 items, loadings 0.32–0.77). (2) Critical thinking and problem-solving skills (CP): Refers to the capacity to think systematically and make decisions by applying logical reasoning to effectively solve problems (3 items, loadings 0.63–0.85). (3) Communication and collaboration skills (CC): Refers to the ability to work well in teams, share responsibility, and lead others ethically and collaboratively to achieve common goals (3 items, loadings 0.40–0.75). (4) IMT: Refers to the ability to analyze, create, and manage media content accurately and ethically across various platforms, using electronic devices and applications effectively while handling informational data appropriately (5 items, loadings 0.31–0.93). (5) Initiative and self-direction skills (IS): Refers to the abilities that help individuals grow and improve in both their personal lives and careers, including effective learning, curiosity, and life skills that contribute to achieving fulfillment, balance, and success (3 items, loadings 0.50–0.95). (6) Social and cross-cultural skills (SC): Refers to the ability to work effectively with people from diverse backgrounds by showing respect and understanding cultural differences (3 items, loadings 0.65–0.94).

#### Confirmatory factor analysis

**Fig. 1.**
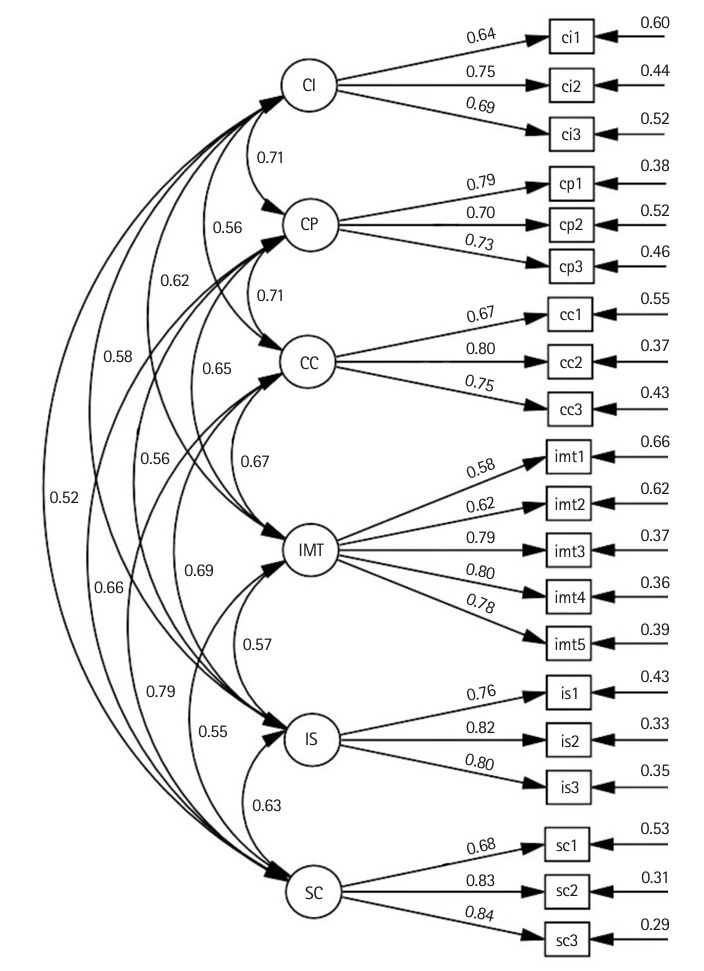
Confirmatory factor analysis model of the 21st Century Skills Assessment Scale. CI, creativity and innovation skills; CP, critical thinking and problem-solving skills; CC, communication and collaboration skills; IMT, information, media, and technology skills; IS, initiative and self-direction skills; SC, social and cross-cultural skills.

CFA confirmed the factor structure using a dataset of 427 subjects. The 21CSAS model demonstrated a good overall fit, explaining approximately 34%–71% of the variance among items. Convergent validity was supported by item loadings ranging from 0.58 to 0.84, while discriminant validity was indicated by correlations between constructs ranging from 0.52 to 0.79 (Tables 1–3, Fig. 1).

#### The 21st Century Skills Assessment Scale for Thai public health students

The 21CSAS demonstrated strong psychometric properties, with notable item-discrimination and high reliability (Table 4). Both EFA and CFA validated the 6-dimensional structure, establishing the 21CSAS as a dependable and effective instrument for assessing 21st-century skills in Thai public health undergraduates.

## Discussion

### Key results

The 21CSAS is a validated tool developed to assess key 21st-century skills among public health undergrad students in Thailand. It evaluates 6 critical skill areas essential for modern public health practice: CI, CP, CC, IMT, IS, and SC. Notably, the 21CSAS has demonstrated both validity and reliability, effectively distinguishing skill levels across different academic years. Analysis reveals that 4th-year students achieve the highest average scores, followed by 3rd-year, 2nd-year, and 1st-year students.

### Interpretation

The 21CSAS framework is vital for equipping students with the skills needed to address complex, real-world challenges. By emphasizing creativity, critical thinking, communication, and collaboration, it prepares individuals to thrive in dynamic professional environments such as public health. These competencies serve as cornerstones for tackling modern health challenges and driving meaningful solutions.

CI is the ability to generate innovative ideas and approach problems with originality. This skill empowers students in public health to think beyond conventional solutions, fostering creativity in addressing pressing issues. CI’s dimensions (ci1, ci2, ci3) reflect its role in encouraging students to develop unique strategies and collaborate effectively. By promoting creativity, students gain the tools to engage with diverse perspectives and offer groundbreaking solutions in evolving contexts.

CP centers on evaluating situations, identifying challenges, and formulating logical solutions. Public health professionals must think systematically and make decisions based on sound reasoning to address multifaceted problems. The cp1, cp2, and cp3 dimensions highlight the importance of structured, critical approaches to problem-solving, which are crucial for navigating complex health scenarios. This skill helps students break down problems and come up with practical solutions.

CC involve exchanging ideas, sharing information, and working cohesively to achieve shared goals. These dimensions (cc1, cc2, cc3) emphasize the necessity of clear communication and teamwork in resolving health-related challenges. Public health relies heavily on coordinated efforts, and the ability to work well in teams is pivotal. The interplay between creativity, problem-solving, and collaboration demonstrates the interdependence of these skills in achieving impactful results.

IMT emphasize the ability to use digital tools, critically assess media content, and ethically apply technology. Public health professionals must be adept at navigating digital platforms, interpreting data, and leveraging technology to design effective interventions. Dimensions imt1 through imt5 reflect the growing reliance on these skills in modern practice, highlighting the need to integrate technological literacy into problem-solving and communication.

IS focus on goal-setting, self-motivation, and independent learning. These skills, represented by is1, is2, and is3, enable students to take charge of their education and remain adaptable in evolving fields. Public health professionals benefit from initiative by continuously updating their knowledge and skills to address emerging challenges. Self-direction fosters resilience and equips students to adapt to various professional demands.

SC emphasize collaboration across diverse backgrounds. In today’s interconnected world, public health professionals must work effectively with individuals from various cultural and social contexts. Measured by sc1, sc2, and sc3, these skills promote inclusivity and adaptability. A connection between initiative and cross-cultural competencies, as self-directed learners are often better equipped to navigate diverse environments and build effective relationships.

By developing these essential skills, the 21CSAS framework prepares public health students to meet the field’s complex and evolving demands. Integrating creativity, problem-solving, communication, technological literacy, self-direction, and cross-cultural collaboration into education enhances job readiness and professional competence. These skills enable students to tackle pressing health challenges, advocate for health equity, and contribute meaningfully to improving global health outcomes. Through this holistic approach, the framework cultivates professionals who are not only capable of addressing contemporary health needs but are also prepared to adapt and thrive in an ever-changing landscape.

### Comparison with previous studies

The 21CSAS improves previous tools by addressing weaknesses in public health education. Previous assessments, such as those validated by Cevik and Senturk [7], often lacked focus on field-specific competencies and did not fully reflect current educational demands. In contrast, the 21CSAS is tailored to these needs, with questions developed from a comprehensive literature review. Additionally, its 5-point Likert scale minimizes response bias and enhances the accuracy of results. The 21CSAS also aligns with findings from Castanheira et al. [8] regarding skill development in achieving educational goals and addresses the need for effective evaluation tools in health professions education, as highlighted by Huang et al. [9].

Public health professionals differ from students in other programs through their focus on community health and preventive measures rather than individual patient care. They are trained to understand population dynamics, social determinants of health, and systemic interventions. This necessitates skills in epidemiology, biostatistics, and health policy analysis, which are less emphasized in traditional medical training.

Additionally, the 21CSAS aligns with the competencies outlined by the Thai Community Health Professions Council, highlighting leadership, advocacy, communication, and ethical practice. By incorporating these core competencies and addressing emerging public health issues such as climate change and pandemics, the 21CSAS effectively prepares future public health leaders to confront pressing global health challenges.

### Limitations

The predominantly female sample (88%) limits generalizability to gender-diverse populations. The cross-sectional design prevents causal inferences on academic year and skill development, complicating long-term impact assessment. Self-reported measures may introduce bias, as students might overestimate their skills. Additionally, the 21CSAS lacks predictive validity testing, leaving its ability to forecast real-world public health performance unexplored.

### Generalizability

Focused on public health undergraduates in Thailand, this study may have broader relevance to similar educational settings in Asia and Europe. The developed assessment scale, based on the P21 framework, can extend beyond public health to fields such as health and social sciences. Adapting this scale across disciplines enables educators to assess and develop essential skills effectively, preparing students for workforce challenges and enhancing curriculum development.

### Suggestions

To improve generalizability, future studies should apply the 21CSAS across diverse disciplines and cultural settings. Combining various data sources (e.g., peer assessments, direct observations, and longitudinal designs) will offer a deeper understanding of students’ 21st-century skills. Educators should analyze assessment data to pinpoint areas of difficulty, which they can address using targeted teaching methods and support. Longitudinal studies testing predictive validity would enhance the scale’s applicability and better prepare students for real-world public health challenges.

### Conclusion

In conclusion, the 21CSAS demonstrates strong psychometric properties, serving as a valuable tool for assessing public health students’ 21st-century skills. The study underscores the importance of collaboration among educational institutions, public health agencies, and communities to align curricula with the evolving demands of the field, ensuring that future professionals are well-prepared to meet emerging challenges and improve the health of diverse populations.

## Figures and Tables

**Figure f2-jeehp-21-37:**
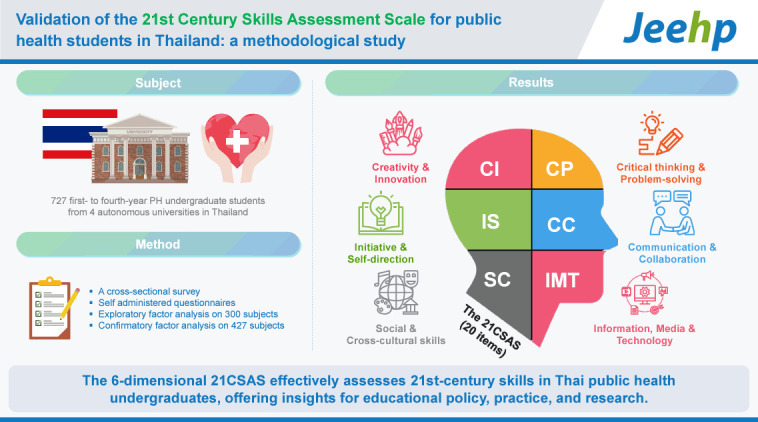


**Table 1.** Goodness-of-fit index parameters: criteria and results of exploratory factor analysis and confirmatory factor analysis models

Indexed	Criteria [11]	EFA	CFA
Normed chi-square (χ^2^/df)	<3	1.99	2.67
Comparative fit index	>0.90	0.97	0.93
Tucker-Lewis index	>0.90	0.93	0.91
Root mean square error of approximation	<0.07	0.06	0.06
Standardized root mean square residual	<0.08	0.02	0.06

EFA, exploratory factor analysis; CFA, confirmatory factor analysis; df, degrees of freedom.

**Table 2.** Exploratory factor analysis and confirmatory factor analysis of the 21st Century Skills Assessment Scale for public health students in Thailand

Factors/items	EFA (n=300) Geomin-rotated loadings^a)^							CFA (n=427)	
	F1	F2	F3	F4	F5	F6	Communality	Loadings	R^2^
CI								AVE=0.72, CR=0.74	
CI1	0.32						0.16	0.64	0.40
CI2	0.77						0.61	0.75	0.56
CI3	0.70						0.66	0.69	0.48
CP								AVE=0.55, CR=0.78	
CP1		0.63					0.43	0.79	0.62
CP2		0.85					0.74	0.70	0.48
CP3		0.63					0.42	0.73	0.54
CC								AVE=0.55, CR=0.79	
CC1			0.40				0.22	0.67	0.45
CC2			0.59			0.31	0.45	0.80	0.63
CC3			0.75				0.41	0.75	0.57
IMT								AVE=0.52, CR=0.84	
IMT1		0.34		0.31			0.24	0.58	0.34
IMT2				0.35			0.20	0.62	0.38
IMT3				0.69			0.52	0.79	0.63
IMT4				0.93			0.88	0.80	0.64
IMT5				0.75			0.60	0.78	0.61
IS								AVE=0.63, CR=0.84	
IS1					0.72		0.52	0.76	0.57
IS2					0.95		0.94	0.82	0.67
IS3					0.50		0.30	0.80	0.65
SC								AVE=0.62, CR=0.83	
SC1						0.65	0.44	0.68	0.47
SC2						0.94	0.88	0.83	0.69
SC3						0.65	0.47	0.84	0.71
Eigenvalues	1.19	1.52	1.07	2.12	1.67	1.73			
% of variance	5.93	7.58	5.35	10.61	8.35	8.64			

EFA, exploratory factor analysis; CFA, confirmatory factor analysis; AVE, average; CR, composite reliability; CI, creativity and innovation skills; CP, critical thinking and problem-solving skills; CC, communication and collaboration skills; IMT, information, media, and technology skills; IS, initiative and self-direction skills; SC, social and cross-cultural skills.

a) Factor loadings <0.30 are not shown; loadings of all items were significant at P<0.001.

**Table 3.** Inter-correlations of scale factors from exploratory factor analysis and confirmatory factor analyses of the 21st Century Skills Assessment Scale for public health students in Thailand

Factors	EFA (n=300) Geomin-factor correlations^a)^						CFA (n=427) factor correlations^a)^					
	CI	CP	CC	IMT	IS	SC	CI	CP	CC	IMT	IS	SC
CI	1.00						1.00					
CP	0.52	1.00					0.71	1.00				
CC	0.39	0.55	1.00				0.56	0.71	1.00			
IMT	0.37	0.46	0.43	1.00			0.62	0.65	0.67	1.00		
IS	0.41	0.29	0.37	0.28	1.00		0.58	0.56	0.69	0.57	1.00	
SC	0.43	0.42	0.53	0.46	0.41	1.00	0.52	0.66	0.79	0.55	0.63	1.00

EFA, exploratory factor analysis; CFA, confirmatory factor analysis; CI, creativity and innovation skills; CP, critical thinking and problem-solving skills; CC, communication and collaboration skills; IMT, information, media, and technology skills; IS, initiative and self-direction skills; SC, social and cross-cultural skills.

a) All values were significant at P<0.05.

**Table 4.** Mean and standard deviation of items and reliability of the 21st Century Skills Assessment Scale (n=727)

Dimensions/items	Mean±SD	CITC
CI (Cronbach’s α=0.72)		
CI1: I have trained myself to take initiative by identifying the problem, which leads to applying creativity in solving it.	3.72±0.70	0.56
CI2: I have trained myself to apply theoretical knowledge and integrate it with real-world problems in the field to create targeted and effective solutions.	3.46±0.73	0.54
CI3: I have trained myself in developing skills in design, creating innovative prototypes, and testing them with sample groups.	3.28±0.82	0.48
CP (Cronbach’s α=0.80)		
CP1: I have trained myself to think logically and reasonably for appropriate situation.	3.96±0.66	0.65
CP2: I have trained myself to think systematically—processes and outcomes—considering various components and variables.	3.74±0.67	0.54
CP3: I have trained myself to think and make decisions to solve problems, taking into account information, resources, options, and the potential impacts of those decisions.	3.91±0.66	0.62
CC (Cronbach’s α=0.78)		
CC1: I effectively collaborate with my team, share responsibilities equally, and lead with integrity to achieve our common goals.	3.73±0.74	0.56
CC2: I have trained myself to work well in groups, such as, contributing ideas, creating together, solving problems together, and sharing responsibility for the outcomes.	4.06±0.71	0.65
CC3: I have trained myself to allocate tasks, assign roles, and take responsibility for assigned tasks, and I can align my contributions with the group’s objectives to achieve shared goals.	4.02±0.71	0.64
IMT (Cronbach’s α=0.84)		
IMT1: I have trained myself to access, analyze, and evaluate information proficiently.	3.76±0.68	0.58
IMT2: I have trained myself to analyze and produce media content, ensuring it is accurate and ethical, regardless of the platform.	3.87±0.70	0.64
IMT3: I have trained myself to efficiently use electronic devices and IT equipment in communication systems.	3.69±0.74	0.57
IMT4: I have trained myself to appropriately use various applications according to their intended purposes.	3.72±0.74	0.58
IMT5: I have trained myself to effectively use informational data.	3.70±0.74	0.59
IS (Cronbach’s α=0.83)		
IS1: I have trained myself to develop life skills that lead to happiness, balance, and success according to my life goals.	3.79±0.77	0.52
IS2: I have trained myself to develop learning skills, to be inquisitive about what I learn, and to re-learn to deepen my understanding of what I think I already know.	3.68±0.70	0.58
IS3: I have taken the initiative to develop the skills necessary for my profession and to guide others.	3.79±0.74	0.63
SC (Cronbach’s α=0.83)		
SC1: I have trained myself to recognize differences in language, religion, lifestyle, customs, traditions, and ethnic characteristics.	3.99±0.70	0.56
SC2: I have trained myself to show acceptance and respect, avoiding judgmental attitudes that devalue others.	4.14±0.68	0.62
SC3: I have trained myself to live and work harmoniously with people from diverse cultural, racial, religious, social, and professional backgrounds.	4.16±0.71	0.66

SD, standard deviation; CITC, corrected item-total correlation; CI, creativity and innovation skills; CP, critical thinking and problem-solving skills; CC, communication and collaboration skills; IMT, information, media, and technology skills; IS, initiative and self-direction skills; SC, social and cross-cultural skills.

## Footnotes

**Data availability**
Data files are available from Harvard Dataverse: https://doi.org/10.7910/DVN/UQP3PI Dataset 1. Research data for the 727 participants’ responses to the 21-item questionnaire.

## Supplementary materials

Supplement 1. Audio recording of the abstract.
